# Enhancing the Resilience of ROS 2-Based Multi-Robot Systems with Kubernetes: A Case Study on UWB-Based Relative Positioning

**DOI:** 10.3390/s25165067

**Published:** 2025-08-14

**Authors:** Jiaqiang Zhang, Xianjia Yu, Tomi Westerlund

**Affiliations:** Turku Intelligent Embedded and Robotic Systems (TIERS) Lab, University of Turku, 20520 Turku, Finland; xianjia.yu@utu.fi (X.Y.); tovewe@utu.fi (T.W.)

**Keywords:** Kubernetes, edge computing, ROS 2, UWB, LSTM, multi-robot relative position

## Abstract

ROS (Robot Operating System) has become the de facto standard in robotics research and development, with ROS 2, in particular, offering enhanced support for real-time communication, distributed systems, and scalable multi-robot applications. These capabilities have driven its widespread adoption across academia, industry, and the open-source community. However, deploying ROS 2 applications across heterogeneous hardware platforms remains a complex task—especially in scenarios that require tightly coordinated, multi-agent systems. In such cases, the failure of a single agent can propagate disruptions throughout the system. A representative example is Ultra-wideband (UWB)-based multi-robot relative localization, where inter-robot dependencies are essential for maintaining accurate relative positioning. While Kubernetes offers powerful features for automated deployment and orchestration, its integration with ROS 2 has not yet been thoroughly evaluated within the context of specific robotic applications. This paper addresses this gap by integrating Kubernetes with ROS 2 in a UWB-based multi-robot localization system, using UWB ranging error mitigation as a representative application. An edge cluster comprising five NVIDIA Jetson Nano devices and one laptop is orchestrated using Kubernetes, with a Jetson Nano node mounted on each robot. We deploy Long Short-Term Memory (LSTM)-based error mitigation modules on the edge nodes and systematically induce failures in various combinations of these modules. The system’s resilience and robustness are then assessed by analyzing position errors under different failure scenarios.

## 1. Introduction

In recent years, the deployment of multi-robot systems has seen a significant increase across various applications, ranging from industrial automation and logistics to search and rescue operations and environmental monitoring. ROS 2 has emerged as the de facto standard for robotic systems, offering a distributed architecture that is particularly well-suited for multi-robot applications. Multi-robot systems have significantly benefited from ROS 2’s features, including real-time communication, modular node management, scalability, and seamless inter-robot data exchange [1]. However, ROS 2 faces several challenges in deployment across heterogeneous hardware platforms, and in certain cases, the sudden failure of individual nodes can negatively impact the overall system performance and stability. Ultra-wideband (UWB)-based relative multi-robot localization is one such case. For instance, the UWB ranging error estimation code can be deployed among different agents with each computing constraints to maximize the computing resources, while each failure may introduce system interruption.The coordination and spatial awareness among robots in such systems are paramount for efficient operation and task fulfillment.

Precise localization serves as a critical foundation in multi-robot systems. UWB-based localization systems offer a cost-effective and relatively accurate performance with the potential of fusing ranging data with odometry and other sensor modalities, forming the backbone of many collaborative robotic applications [2]. However, UWB measurements are susceptible to various sources of error, including noise, non-line-of-sight (NLOS) interference, and multipath effects, which can compromise overall system accuracy. Recent studies have shown that integrating Long Short-Term Memory (LSTM) with UWB-based localization systems significantly improves accuracy [2,3]. The UWB ranging error estimation code can be distributed across multiple agents with varying computational capabilities to optimize the use of available computing resources; however, the failure of any individual agent can lead to severe degradation in system accuracy and reliability. Ensuring the resilience and robustness of UWB-based systems in distributed ROS 2 multi-robot systems remains an open challenge.

Meanwhile, containerization has revolutionized the deployment and management of applications by encapsulating software and its dependencies into isolated units called containers, with Docker being a leading platform in ensuring consistent runtime environments across different systems [4,5]. Kubernetes (and lightweight distributions such as K3s) automates deployment, monitoring, and self-healing of containerized applications, potentially enabling multi-robot systems to recover from software node failures with minimal human intervention. Existing studies explore the transformative impact of containerization and orchestration in robotic systems [6,7,8]. However, there is a notable lack of practical investigations into how the deployment of Kubernetes with ROS 2 influences the performance of specific robotic tasks such as multi-robot relative localization.

To address these issues, this paper introduces an innovative approach to deploying a UWB-based multi-robot relative positioning system on a Kubernetes edge cluster with ROS 2. The core contribution of this study lies in the implementation of the aforementioned system and demonstration of its impact on the performance in a relative localization application, leveraging edge computing in robotics. Specifically, we showcase how the deployment of complex algorithms including LSTM for UWB ranging error correction and a particle filter for position estimation, using Kubernetes and ROS 2 on an edge cluster, can significantly enhance the robustness and resilience of multi-robot systems.

The main contributions of this paper are as follows:

- **Kubernetes-orchestrated ROS 2 edge architecture.** We design and implement a K3S-based orchestration of containerized ROS 2 nodes for a multi-robot relative localization task, detailing deployment descriptors and node placement (worker/master) relevant to resource-constrained robots.
- **UWB error mitigation at the edge.** We integrate an LSTM-based ranging error estimator as distributed ROS 2 nodes on each robot, feeding a particle filter relative pose estimator.
- **Fault injection evaluation of resilience.** We induce controlled LSTM node failures (F1–F5 scenarios) during operation as well as measure localization performance and recovery, showing graceful degradation compared to a no-LSTM baseline (NOLSTM) and rapid restoration via Kubernetes self-healing.

The rest of this paper is organized as follows: Section 3.1 describes the system architecture. Section 3.2 details the resilient and robust multi-robot relative positioning deployment, covering the Docker registry, particle filter Node, LSTM Nodes, and K3S Edge Cluster. Section 4 presents the experimental results, and Section 5 concludes the paper, summarizing the findings and suggesting directions for future research.

## 2. Background and Related Works

### 2.1. Conceptual Background

**ROS 2 for distributed robotics:** ROS 2 provides a modular, distributed computation model in which nodes exchange typed messages over topics and services. Built atop DDS, ROS 2 supports discovery across multiple hosts, QoS policies, and real-time-oriented execution, enabling multi-robot data exchange and modular deployment across heterogeneous computing [1,9].

**Kubernetes and K3S for edge orchestration:** Kubernetes, an open-source container orchestration platform, automates the deployment, scaling, and management of these containerized applications, providing a robust framework for complex, distributed systems [1,10]. K3s is a lightweight distribution designed for resource-constrained devices while preserving core orchestration semantics, making it suitable for edge clusters comprising embedded GPUs and laptops [11].

### 2.2. Related Works

Kubernetes-based solutions like K3S are gaining traction for their efficiency in resource-constrained environments [11]. Several studies have explored the integration of containerization technologies in robotics, emphasizing modularity, portability, and ease of deployment. For instance, [6] introduced a scalable Kubernetes-based framework for Internet of Drones (IoD), while [12] utilized KubeEdge to orchestrate ROS 1-based applications. Lumpp et al. introduced a container-based methodology for deploying ROS 1 on edge-cloud architectures and explored mixed-criticality orchestration to enhance reliability in autonomous system [7,13,14]. Other works, such as [8,15], demonstrated edge computing architectures using Kubernetes for model predictive control (MPC) in ROS 1 aerial robots, enhancing control accuracy and responsiveness. In the work [16], the authors compared Docker- and Kubernetes-based edge architectures in MPC for aerial robots. Additionally, research has evolved towards more lightweight models, like unikernels [17], and integrated blockchain with multi-access edge computing to address challenges of decentralized control and data security [18]. Outside the robotics domain, cutting-edge edge computing research is highly relevant for orchestration and resource management. For example, Liu et al. [19] propose a multi-agent deep reinforcement learning framework for distributed computation offloading in wireless-powered MEC networks, which optimizes energy usage while meeting latency constraints.

These studies underscore the transformative impact of containerization and orchestration in enhancing scalability, efficiency, and reliability in robotic systems. However, these works primarily concentrate on system design, with most implementations using ROS 1 and only a limited number utilizing ROS 2. Furthermore, there is a notable lack of practical investigations into how the deployment of Kubernetes with ROS 2 influences the performance of specific robotic tasks such as multi-robot relative localization.

## 3. Resilient Multi-Robot Relative Positioning Using Kubernetes and ROS 2

### 3.1. System Architecture

While containerization and orchestration technologies such as Docker and Kubernetes have transformed software deployment by offering modularity, portability, and scalability, their application in robotics presents unique challenges. Robotic systems are often resource-constrained, operate in dynamic environments, and require real-time responsiveness, which complicates the direct adoption of traditional container solutions. Furthermore, orchestrating containerized robotic applications across distributed systems—often involving edge, cloud, and mixed-criticality components—demands optimized frameworks that can balance resource efficiency, computational loads, and communication reliability. These challenges necessitate tailored approaches to fully realize the potential of container technologies in the context of robotics.

**Figure 1:**
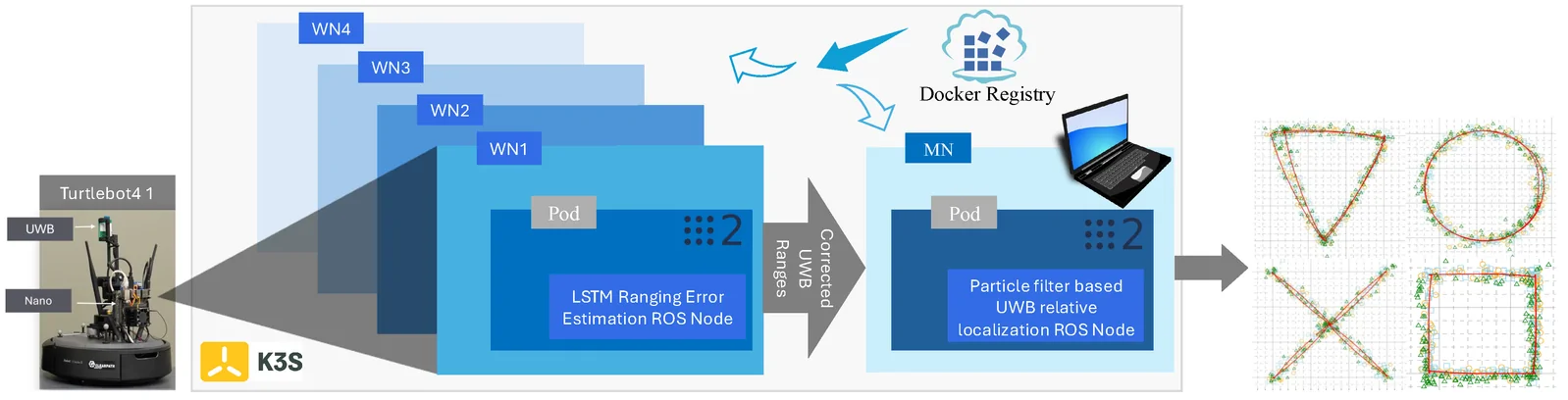
Illustration of the proposed system architecture. A Worker Node (WN) and a Master Node (MN) are managed by K3S. UWB measurements from the robots (WNs) are refined via an LSTM-based Ranging Error Estimation ROS 2 Node and sent to the MN, where a Particle Filter-based ROS 2 Node processes them for relative localization. The ROS 2 nodes run in a container, Pod. The LSTM ranging error estimation programs and particle filter programs are pulled from a private Docker registry to WNs or the MN. This setup enhances the resilience and robustness of UWB multi-robot indoor positioning.

Figure 1 illustrates the system architecture of the proposed UWB-based multi-robot relative localization system deployed on a K3S edge cluster. The system is designed to leverage sensor fusion techniques and distributed computing to achieve resilient and accurate localization in resource-constrained environments, such as those typical of multi-robot systems operating in the field. The architecture integrates UWB ranging for distance measurement, LSTM networks for UWB error estimation, and particle filter-based algorithms for precise relative localization.

As shown in Figure 1, each Turtlebot4 is equipped with one UWB transceiver and a Nvidia Jetson Nano, which collects UWB ranging data and runs a LSTM ROS 2 node to estimate and correct errors. Additionally, each LSTM node processes the UWB ranges associated with its respective transceiver. Overlapping ranges are manually removed to prevent duplicate corrections of the same measurement. These LSTM nodes are deployed on Jetson Nano as worker nodes (WNs) within the K3S cluster, publishing corrected UWB ranges. A particle filter ROS 2 node, running on a laptop as the master node (MN), processes the corrected UWB data to compute the relative positions of the robots. Pods are the smallest deployable units of computing in a Kubernetes cluster. Docker container images are pulled from a Docker registry to ensure consistent deployment across the K3S cluster. The figure also shows the final robot trajectories, which demonstrate the system’s ability to achieve accurate localization. The K3S edge cluster ensures fault tolerance and efficient management of the containerized nodes.

### 3.2. System Deployment

The deployment of the system on the K3S cluster involves creating and managing containers for the LSTM nodes and the particle filter node. Kubernetes automates the deployment, scaling, and management of these containers to ensure efficient operation and resource utilization.

#### 3.2.1. Docker Registry

**Figure 2:**
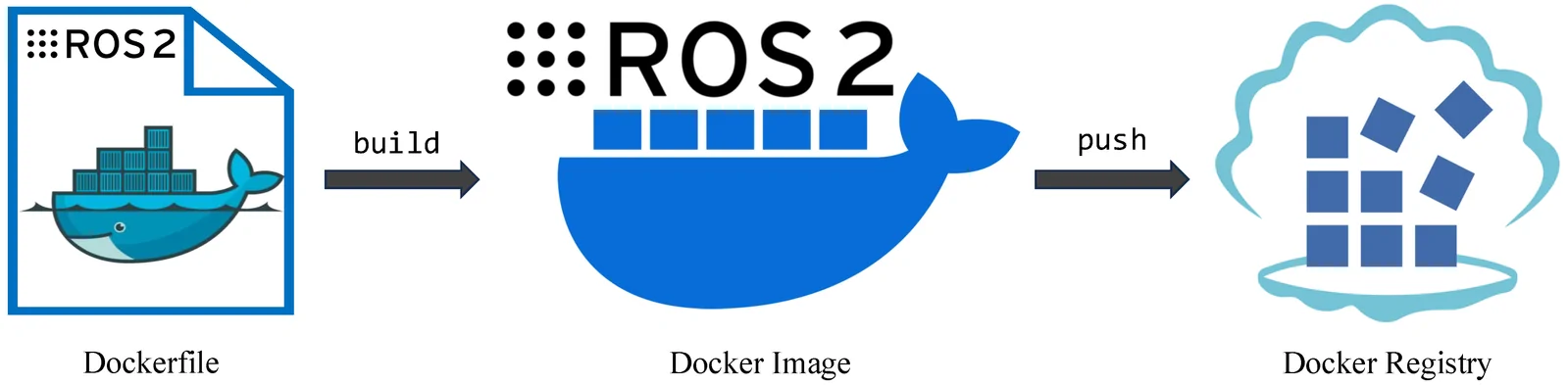
Containerized ROS 2 application workflow.

To streamline the deployment process, Docker images for the LSTM and particle filter applications are created and stored in a private Docker registry. The workflow of containerization is shown in Figure 2. This registry serves as a centralized repository for all Docker images, ensuring secure and efficient distribution of containerized applications across the edge cluster.

#### 3.2.2. Particle Filter Node

Since this paper emphasizes systematic implementation, the individual particle filters were adopted from our previous work, where detailed information and code are available [2]. Briefly, each particle filter uses the robot’s odometry to propagate its state, and updates the state using range measurements from UWB transceivers as observations. It is worth noting that the state in this work includes only the *x* and *y* coordinates of each robot, with all robots sharing the same local reference frame.

The particle filter node is containerized using Docker and deployed on the K3S cluster. This node subscribes to the UWB ranges published by the UWB transceivers or the corrected UWB ranges published by the LSTM nodes and uses a particle filter algorithm to estimate the relative positions of the robots. The estimated positions are then published to a ROS topic. The implementation of the particle filter node is based on the methodology described in [2]. The deployment setup of the particle filter node is shown as follows:

apiVersion:  apps/v1
kind:  Deployment
metadata:
    name:  pf-ros2-deployment
    labels:
        app:  pf-ros2
spec:
    selector:
        matchLabels:
            app:  pf-ros2
    template:
        metadata:
            labels:
                app:  pf-ros2
        spec:
            containers:
                − name:  pf-ros2
                  image:  pf-ros2: ros2core

#### 3.2.3. LSTM Nodes

The computational resource is limited in an edge scenario, especially for machine learning applications. To simulate such cases, we divide the LSTM calculating process into 5 nodes. The LSTM nodes are responsible for processing UWB-ranging data to correct measurement errors. Each LSTM node runs in its own container, subscribing to UWB data and publishing corrected ranges to a ROS topic. The LSTM network is based on the one from the work [2]. The LSTM network consists of two LSTM layers with 50 units each, followed by dropout layers with a 30% rate to reduce overfitting. A final dense layer outputs a single value representing the ranging error. This architecture captures temporal dependencies and predicts the error from input sequences. The model was trained using a separate dataset collected within the same facility. Its effectiveness in mitigating ranging errors was validated in the work, where more detailed information and codes are provided [2]. The deployment setup for the LSTM nodes is illustrated as follows:

kind:  Deployment
metadata:
    name:  uwb-lstm-deploymentxxx
    labels:
        app:  uwb-lstmxxx
spec:
    selector:
        matchLabels:
            app:  uwb-lstmxxx
    template:
        metadata:
            labels:
                app:  uwb-lstmxxx
        spec:
            containers:
                −  name:  uwb-lstm-ros2core-xxx
                    image:  uwb-lstm-ros2core:xxx

#### 3.2.4. K3S Edge Cluster

The K3S cluster orchestrates the deployment and management of the LSTM and particle filter nodes. The cluster comprises five NVIDIA Jetson Nano devices, each mounted on a Turtlebot4 platform and functioning as worker nodes, and a laptop serving as the master node. This configuration ensures efficient resource utilization, fault tolerance, and scalability. The K3S cluster automatically handles node failures and load balancing, maintaining the system’s operational integrity.

### 3.3. Evaluation Metrics

As our primary focus is on examining the impact of Kubernetes deployment with ROS 2 on the performance of multi-robot relative localization, we utilize the Absolute Pose Error (APE) as a metric to evaluate the deployment’s effectiveness. An OptiTrack mocap system was utilized to provide the ground truth for the relative position results. The tool utilized for the computation of APE values is the EVO (https://github.com/MichaelGrupp/evo (accessed on 30 December 2024)) in Python.

### 3.4. Experimental Setup

#### 3.4.1. Overall Setting

To thoroughly evaluate the resilience and robustness of the proposed multi-robot relative localization system, we conducted the experiment in a controlled indoor environment with a series of experimental scenarios. These scenarios are intended to simulate various failure conditions of the LSTM nodes, thereby assessing the system’s fault tolerance and resilience. The experiments are conducted using four Turtlebot4 platforms, and the settings are defined in Table 1. We executed six failure injection scenarios plus one baseline (NOLSTM). Each scenario is defined by (i) the number of LSTM pods that are running at start-up and (ii) how many of those pods we deliberately terminate at t = 15 s. The PF pod (hosted on MN) remains operational in every scenario. Immediately after a termination, K3S attempts to restart the killed pods.

In these setting scenarios, the failures of the LSTM nodes are manually induced by stopping the LSTM pods during the experiment. The K3S orchestration system automatically detects these failures and attempts to recover by restarting the failed LSTM pods. This process allows for an in-depth analysis of the system’s fault tolerance, resilience, and the impact of LSTM node failures on overall localization performance.

In a multi-robot system, a key challenge is maintaining clear and reliable communication between robots, as each robot needs to be uniquely identifiable to avoid conflicts and miscommunication. Traditional ROS 2 communication methods can become complex and challenging in environments with wireless-connected multiple robots operating simultaneously as it may bring the data flooding issue to the network. Meanwhile, Zenoh showed better communication performance in a multi-host ROS 2 system under a Wi-Fi network setup [9]. To address the communication challenges in the multi-robot system, we configured the system following the approach outlined in [2].

#### 3.4.2. Software Information

The system was implemented on Ubuntu 20.04, utilizing ROS 2 Galactic in Python 3.8. The LSTM network was developed based on PyTorch framework. K3S v1.27.4 was used for the deployment.

#### 3.4.3. Hardware Information

As we mentioned, the robot as a work node is Turtlebot4 mounted with Nvidia Jetson Nano with 4 GB RAM GPU as shown in Figure 1. The master node is a laptop with AMD Ryzen 7 5800h CPU, 16 GB RAM. The UWB transceiver is from Qorvo. Additionally, the mocap system used in the setup for ground truth relative localization results is from OptiTrack, covering an area of 9 m × 9 m × 5 m.

## 4. Experimental Results

To assess the proposed system’s resilience and fault tolerance, we conducted experiments where LSTM pods were manually stopped mid-operation, and the K3S orchestration system automatically restarted them. Following the experiments, we collected relative localization results and performed a comprehensive analysis.

**Figure 3:**
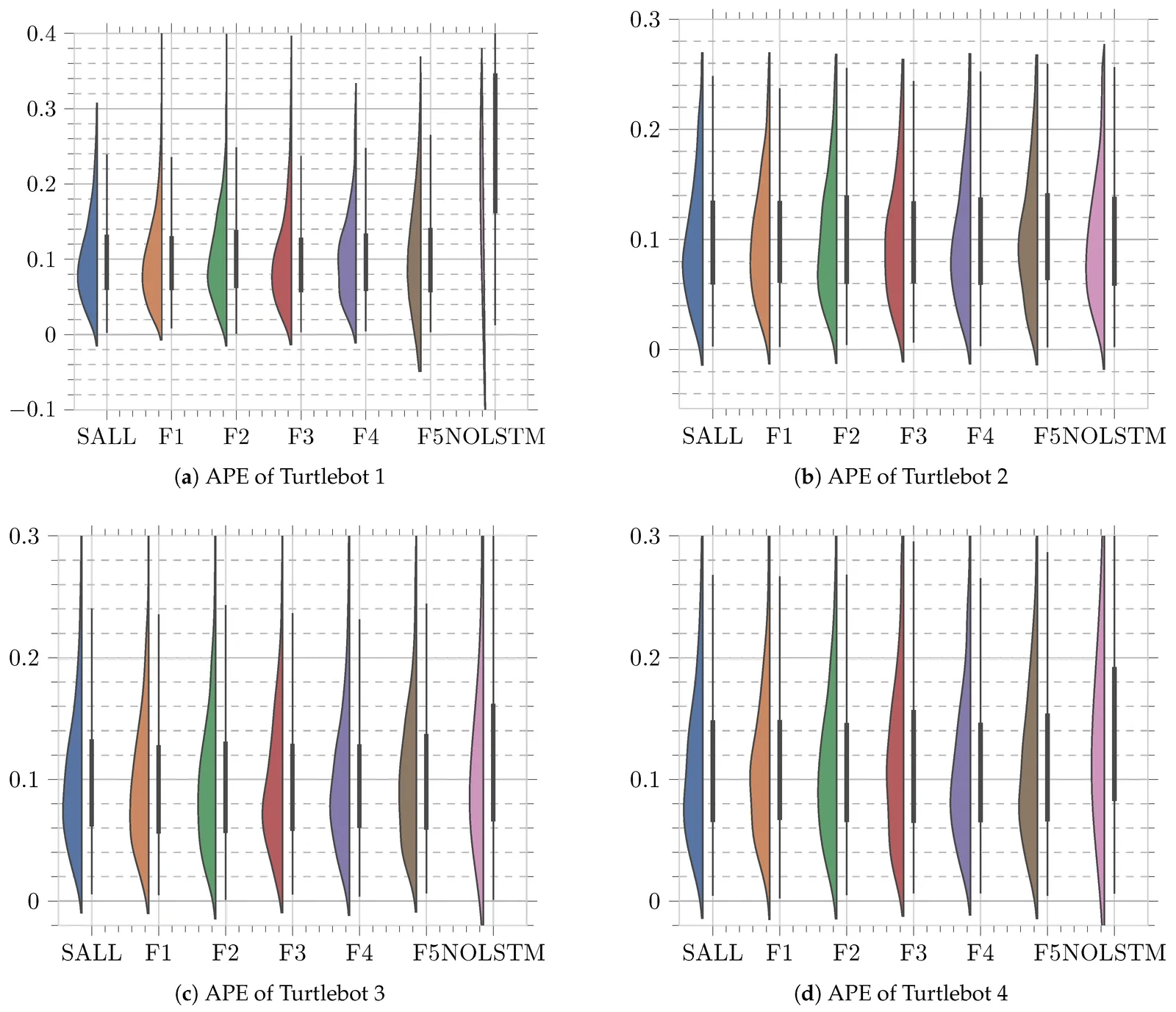
The Absolute Pose Error (APE, unit: m) values of the moving robots under different experimental settings shown in Table 1—Turtlebot 1 to Turtlebot 4—are presented sequentially from left to right.

The APE values of the relative position results under various experimental settings, as detailed in Table 1, are presented in Figure 3. From the figure, it is evident that even in scenarios where LSTM node failures occur, regardless of the number of nodes that fail, the positioning accuracy remains comparable to the scenario without any failures. Furthermore, the accuracy is significantly better than in the case where no LSTM nodes are used. The violin plots show the distribution of the APE values. To provide a concrete comparison of the value differences, Table 2 presents the numerical results for the Root Mean Square Error (RMSE) and its Standard Deviation (STD). These metrics further confirm the observed trends. The results from the scenario without any LSTM nodes typically exhibit higher RMSE and STD values, indicating greater errors and fluctuations in the positioning outcomes. In contrast, the proposed system demonstrates steady performance, maintaining accuracy and reliability even in the presence of node failures.

**Figure 4:**
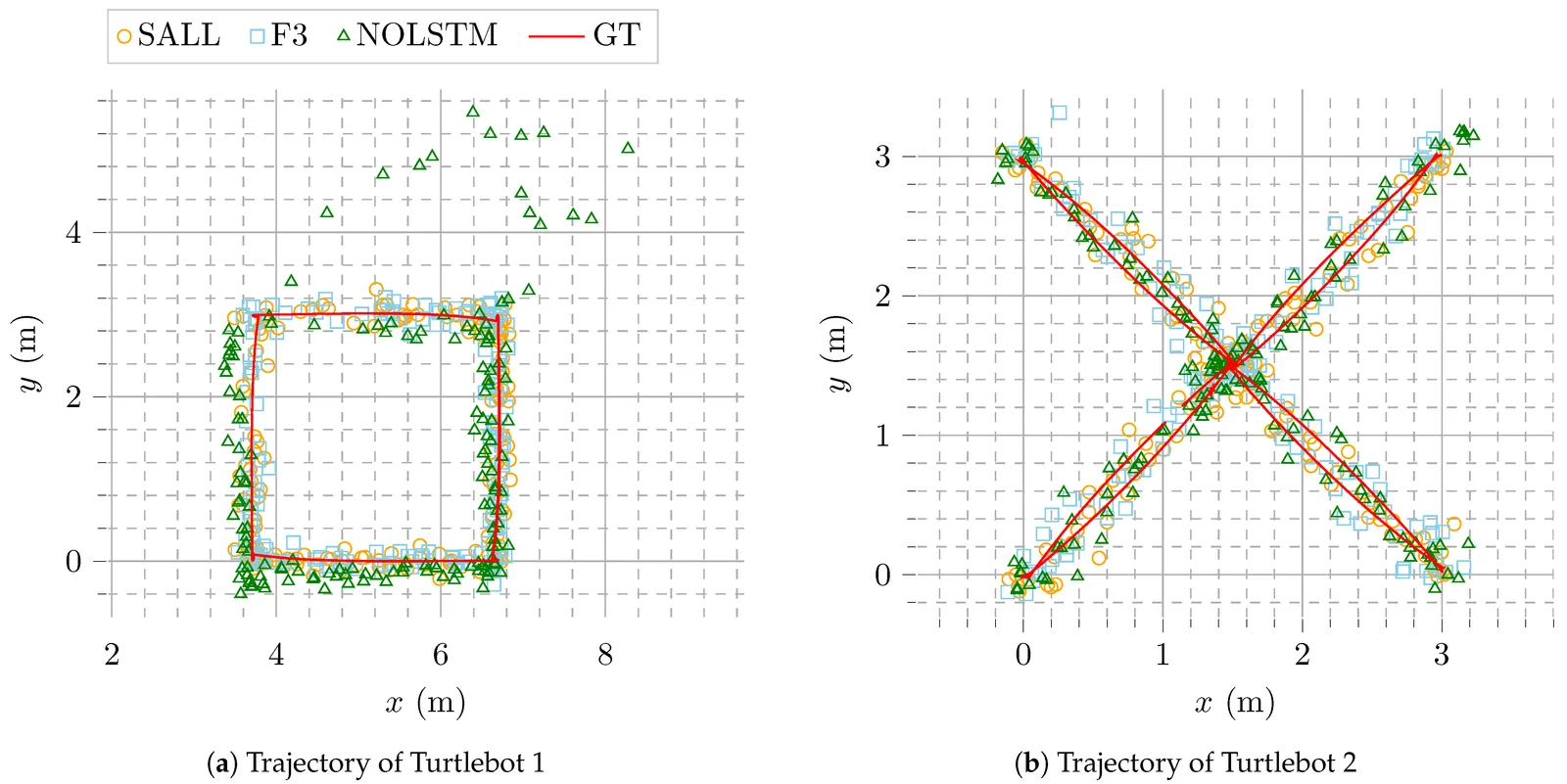

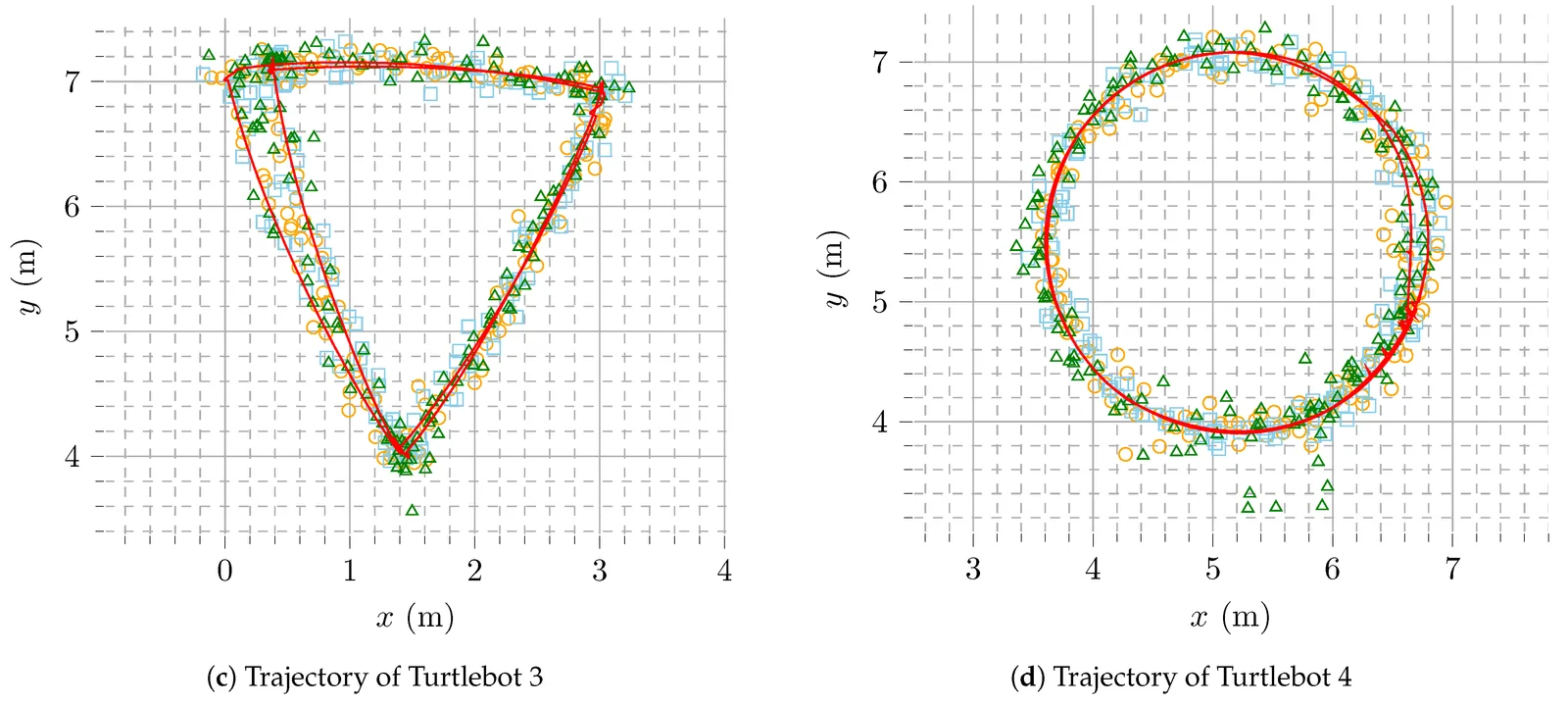
Trajectories of moving robots under different experimental settings, Turtlebot 1 to 4, from left to right.

It is worth noting that the proposed system exhibits resilience by maintaining reasonable accuracy even under multiple LSTM node failures. The trajectories of the Turtlebot (Figure 4) further confirm that position estimations remain largely consistent despite the failure of LSTM nodes, highlighting the system’s resilience under fault conditions. As the accuracy did not significantly change despite the number of failed LSTM nodes, Figure 4 presents the trajectories for scenarios where all LSTM nodes failed and where three LSTM nodes failed. This provides a clearer visualization of the trajectory results under different conditions. The trajectories clearly illustrate that the proposed system effectively mitigates positioning drift observed in the absence of LSTM nodes. For instance, in the first and fourth trajectories in Figure 4, the system maintains trajectory consistency comparable to scenarios where all LSTM nodes function correctly, highlighting its ability to preserve accuracy despite node failures.

## 5. Conclusions and Discussion

### 5.1. Conclusions

In conclusion, this work demonstrates a novel approach to enhancing the resilience of UWB-based multi-robot relative localization systems by deploying LSTM-based error correction and particle filter algorithms on a Kubernetes edge cluster with ROS 2. The comprehensive experimental results show that the system maintains consistent localization accuracy despite multiple node failures, with Kubernetes effectively restoring disrupted nodes to minimize the impact of LSTM node failures. This resilience is evident even under scenarios with a higher number of failed LSTM nodes. These findings emphasize the effectiveness of Kubernetes with ROS 2 in enhancing resilience and robustness in multi-robot systems, offering a fault-tolerant solution for reliable operations in resource-constrained environments and enabling further robotic application deployments with Kubernetes and ROS 2.

### 5.2. Discussion

Future work could investigate the integration of additional sensor modalities, such as LiDAR, cameras, or inertial measurement units (IMUs), to further enhance system robustness. These sensors, when fused with UWB data, could provide complementary information to overcome limitations in challenging environments, such as those with significant NLOS interference. Moreover, the dynamic deployment and management of sensor-dependent algorithms using Kubernetes highlight the importance of scalable orchestration frameworks in handling computationally intensive multi-sensor fusion tasks.

Another promising direction lies in optimizing the system for resilience-critical applications where sensor reliability is paramount, such as autonomous navigation in GNSS-denied environments or cooperative localization in multi-UAV systems. Incorporating blockchain technology could also strengthen data security by ensuring the integrity and trustworthiness of sensor data shared among robots, without compromising system resilience.

## Figures and Tables

**Table 1:** Abbreviations and descriptions of experimental setting scenarios.

Scenario	LSTM Pods Running at t=0 s	Failure Event at t=15 s	LSTM Pods Running 5 s After Failure	Purpose
NOLSTM	0	—	0	Pure UWB + PF baseline (no correction)
SALL	5	none	5	Ideal case: full correction, no faults
F1	5	kill 1 pod	5 (after restart)	Single-node fault, tests self-healing
F2	5	kill 2 pods	5 (after restart)	Dual-node fault, moderate stress
F3	5	kill 3 pods	5 (after restart)	Majority failure, high stress
F4	5	kill 4 pods	5 (after restart)	Only one pod initially survives
F5	5	kill all 5 pods	5 (after restarts)	Worst case: complete outage, full recovery required

**Table 2:** Root Mean Square Error (RMSE, unit: m) and its corresponding Standard Deviation (STD, unit: m) for different moving robots (TB represents Turtlebot) across various experimental settings.

Scenarios	RMSE/STD			
	**TB1**	**TB2**	**TB3**	**TB4**
SAll	(0.121/0.064)	(0.122/0.064)	(0.117/0.057)	(0.133/0.070)
F1	(0.116/0.058)	(0.118/0.058)	(0.113/0.057)	(0.132/0.065)
F2	(0.120/0.060)	(0.123/0.062)	(0.116/0.059)	(0.136/0.074)
F3	(0.118/0.062)	(0.125/0.067)	(0.113/0.056)	(0.138/0.071)
F4	(0.118/0.058)	(0.121/0.061)	(0.116/0.059)	(0.131/0.067)
F5	(0.230/0.195)	(0.122/0.060)	(0.118/0.058)	(0.136/0.070)
NOLSTM	(0.661/0.528)	(0.134/0.076)	(0.188/0.127)	(0.255/0.186)

## Data Availability

The original contributions presented in this study are included in the article. Further inquiries can be directed to the corresponding author.
